# A multinational survey of patient utilization of and value conveyed through virtual symptom triage and healthcare referral

**DOI:** 10.3389/fpubh.2022.1047291

**Published:** 2023-02-02

**Authors:** George A. Gellert, Piotr M. Orzechowski, Tim Price, Aleksandra Kabat-Karabon, Jakub Jaszczak, Natalia Marcjasz, Agata Mlodawska, Aleksandra K. Kwiecien, Piotr Kurkiewicz

**Affiliations:** ^1^Impact/Value Demonstration, Infermedica, San Antonio, TX, United States; ^2^Infermedica, Wroclaw, Poland; ^3^Infermedica, London, United Kingdom

**Keywords:** virtual clinical triage/referral, symptom checker, preclinical triage, digital triage, digital front end

## Abstract

**Objective:**

To describe the use patterns, impact and derived patient-user value of a mobile web-based virtual triage/symptom checker.

**Methods:**

Online survey of 2,113 web-based patient-users of a virtual triage/symptom checker was completed over an 8-week period. Questions focused on triage and care objectives, pre- and post-triage care intent, frequency of use, value derived and satisfaction with virtual triage. Responses were analyzed and stratified to characterize patient-user pre-triage and post-triage intent relative to triage engine output.

**Results:**

Seventy-eight percent of virtual triage users were female, and 37% were 18–24 years old or younger, 28% were 25–44, 16% were 45–54, and 19% were 55 years or older; 41.2% completed the survey from the U.S., 12.5% from the U.K., 9.1% from Canada, 5.6% from India, 3.8% from South Africa. Motivations were to determine need to consult a physician (44.2%), to secure medical advice without visiting a physician (21.0%), and to confirm a diagnosis received (14.2%). Forty-three percent were first time users of virtual triage, 36.6% utilized a triage engine at least once every few months or more often. Pre-triage, 40.5% did not know what level of healthcare they were planning to utilize, 33.9% stated they intended to seek a physician consultation, 23.7% engage self-care and 1.8% seek emergency care. Virtual triage recommended 56.8% of patient-users consult a physician, 33.8% seek emergency care and 9.4% engage self-care. In three-fourths, virtual triage helped users decide level of care to pursue. Among 74.1%, triage recommended care different than pre-triage intentions. Post-triage, those who remained uncertain of their care path decreased by 25.4%. Patient-user experience and satisfaction with virtual triage was high, with 80.1% stating that they were highly likely or likely to use it again, and interest in and willingness to use telemedicine doubled.

**Conclusion:**

Virtual triage successfully redirected patient-users who initially planned to seek an inappropriate level of care acuity, reduced patient uncertainty of care path, and doubled the percentage of patients amenable to telemedicine and virtual health engagement. Patient-users were highly satisfied with virtual triage and the virtual triage patient experience, and a large majority will use virtual triage recurrently in the future.

## Introduction: Superseding patient internet searches with evidence-based clinical triage to convey needed actionable medical information

The public increasingly seeks healthcare information, primarily through the internet, to make informed decisions about their health. Nearly seven percent of daily Google searches concern healthcare topics—representing over 1 billion searches per day (1). Virtual, online or digital patient triage engines, commonly referred to as “symptom checkers,” are being positioned on the digital front door of healthcare systems at the onset of healthcare seeking in order to serve as effective digital guides that connect patient end users to the right level and acuity of healthcare services, potentially optimizing utilization of the appropriate—most clinically-effective and cost-effective—site of healthcare service delivery. Virtual triage can be applied across the spectrum of healthcare needs and concerns, from clinical issues that can be resolved with self-care in the home through primary care to emergent care in an emergency department (ED) or requiring ambulance transport to an ED.

Patient uncertainty or anxiety can lead to selecting a level of healthcare in excess of that clinically needed, generating unnecessary high acuity care with the associated avoidable costs, stress, and resource use. The average cost of treating primary care conditions in an emergency department in the US is $530–2,032 (2, 3). In comparison, a visit with the same clinical presentation to a primary care physician's office or an urgent care center costs $167 or $193, respectively (2, 4). At the other end of the spectrum of clinical severity, patients with clinical conditions requiring immediate acute care who might not seek appropriate emergent care based on uninformed self-assessment and generic information available on the internet, represent a risk of avoidable serious morbidity, poor clinical outcomes and utilization of a level of care acuity in excess of that which would have been required if the patient had sought and received less acute care earlier (5, 6).

In response, as healthcare delivery organizations bring more of their services online in the form of patient portals and telemedicine, digital front doors with virtual triage are being implemented that can serve as the first touchpoints for patient-users (or family members) (7). Thus, individuals currently using a smartphone, tablet or laptop to assess their symptoms on popular browsers of essentially random internet healthcare content can instead use a systematic virtual triage engine as a physician-certified digital tool for preliminary assessment of their symptoms, one that also conveys reliable, actionable clinical care guidance. Virtual triage is a digital technology that patients/families can engage 24/7/365 and is accessible from any device with internet connectivity to assist in evaluating their symptoms and determining the appropriate care needed.

Virtual triage is superior to a generic internet browser search because patients are evaluated and conveyed a triage level using an evidence-based set of clinical algorithms advising if and how quickly they should consult with a physician or other care provider, or if they should instead engage self-care. Patients can verify their health status and care needs in just a few minutes, with information that has been extensively vetted/validated by physicians, and patient access is not limited to usual physician's office or working hours or by requiring travel to and possibly long waits in an ED. Large public health systems such as the British National Health Service and Healthdirect in Australia have adopted virtual triage in recent years (8).

Often individuals are uncertain if their symptoms warrant medical attention, and if their condition worsens, are unsure of the next steps (9). Patients may struggle to navigate the complexity of available medical services—which can be exacerbated given high variability in cost and health insurance coverage (10, 11). Virtual triage used at the beginning of a patient's journey can help provide patients with guidance across available and clinically suitable healthcare services (8). If integrated with a healthcare delivery system, virtual triage can also propose very specific services and care settings available within the user's network that have been informed by the patient's clinical presentation and needs (8). This can both accelerate obtaining needed acute care and reduce unnecessary healthcare seeking in settings of greater acuity, complexity and cost than the individual's illness and chief complaints clinically warrant.

Along with improving the experience of a single patient and their care outcomes, the aggregate de-identified data/information collected and processed by virtual triage engines can discern population-level incidence trends and utilization statistics, which can be of value for healthcare delivery system planning, service delivery improvement and capacity building. By reducing patient disorientation or lack of familiarity with the healthcare delivery system, along with the stress and anxiety associated with seeking healthcare when acutely ill, virtual triage can improve patient satisfaction and retention through greater personalization of service delivery. While a central value conveyed by virtual triage is enabling patients to rapidly secure the appropriate level of care needed for their clinical concern, effective clinical triage can also potentially reduce patient volume pressure in high demand service lines, such as the ED, improve the efficiency/appropriateness of clinical staffing, and possibly reduce clinician stress and dissatisfaction resulting when substantial percentages of patients presenting during high volume ED shifts do not warrant emergent care. For hospital and other care delivery systems, virtual triage can contribute to efforts to reduce patient leakage and retain patients' engagement within a system network. Table 1 summarizes key differentiating features of virtual triage vs. general internet browser searches for meeting public medical information and guidance needs.

**Table 1:** Differentiation of virtual triage engines from generic internet medical content searches.

**Feature**	**Internet search engines**	**Evidence-based virtual triage**
Input data	Single key words/phrases	Responses to highly specific clinical queries about multiple coexisting symptoms (including risk factors) through algorithms informed by artificial intelligence
Information results produced	Random information, unrelated to and uninformed by the user's clinical case and information	Evidence-based health assessment based on patient-user clinical and demographic data
Search engine optimization (SEO)	Based on the most popular searches and non-clinical SEO optimization	SEO-independent, focused on and informed by individual user case and clinical information/symptoms
Quality of assessment and clinical guidance	Difficult to assess, especially without medical training or substantial knowledge	Confirmed by specialty board-certified and licensed physicians, as part of a defined and audited evidence-based quality management system
Time required to provide patient actionable information and clinical guidance	Varied, depending on the quality of results produced by search and the number of topics/issues searched	A few minutes, after which specific and clinically actionable recommendations are presented
Next healthcare seeking actions	Unknown and not informed by clinical expertise	Care recommendations provided and linked with/accelerated to specific appropriate and available clinical care services
Objectivity of care recommendations	Mixed, frequently built around worst possible clinical scenarios found online (negative selection bias)	Objective, evidence-based and conveying reliable clinical guidance

## Materials and methods

### Study objectives

The purpose of this online survey was to better understand and characterize users of a leading triage engine, including demographic variables. In addition, the survey analyses sought to assess what kinds of personal care seeking objectives and intentions patient-users have for a triage engine, including motivation and understanding of their care needs. The survey sought to assess alignment between patient-user self-perception of healthcare need and that recommended by the triage engine. Information on patterns of triage engine utilization was also collected and evaluated, along with future intent to use virtual triage and user satisfaction with virtual triage.

### Study design, setting and description of intervention/virtual triage engine utilized

An online survey was conducted among all patient/consumer users of the Symptomate online clinical triage application. Symptomate is a stand-alone virtual triage engine from Infermedica not attached to a specific health system. It is engaged only by users who are independently seeking a virtual triage tool or symptom checker. The application is designed for patients and estimates the probability of specific diseases based on its triage engine, issuing recommendations for further treatment or contact with a healthcare professional, or self-care and monitoring, as needed. The triage engine is available through the Infermedica website and as a mobile application downloadable from the Apple Store and Google Play. The survey was only available to those accessing Symptomate from a web browser, which comprises 90%+ of virtual triage users.

The virtual triage process does not constitute medical consultation, and its results are not diagnostic, providing information and guidance only. Users are advised not to use virtual triage in an acute emergency, and if a threat to life exists, to call an ambulance immediately. A triage interview can be completed by the user themselves or on behalf of a third party, such as a child (the virtual triage engine includes pediatric conditions content and assessment) and resembles or simulates a conversation with a medical professional. At the start and end of the interview patient-users are asked about their care intention e.g., will they pursue self-care or go to a physician's office or an ED. After gathering basic demographic information (gender, age), subsequent steps ask users to provide information about symptoms present, severity and duration, disease risk factors, medical and travel history. This can be done from a dropdown list, by clicking on a body part of a displayed avatar and selecting a symptom(s) from a list, or by entering a symptom in a search bar. The triage engine then asks questions specific to the case, based on the information gathered.

The triage engine carefully analyzes the user's responses and displays a summary that may include an overall health assessment describing which symptoms require urgent medical attention, and what the patient should consider doing next. The engine's analytics include over 800 conditions, almost 1,500 symptoms, and over 200 disease risk factors. It leverages state-of-the-art technologies, including artificial intelligence, machine learning, and natural language processing, joined with medical evidence to understand and process the symptoms reported by the patient, to suggest the most probable conditions matching their symptoms, and to share the most clinically suitable, safe and effective care pathway, from self-care to visiting a physician's office for consultation or proceed to an emergency department. Another section displays the likely causes of the reported symptoms, allowing the user to click on a specific result for details and to review information supporting the identification of probable conditions. Based on the health assessment, if not requiring an ED visit, a recommendation for self-care or to consult a healthcare professional (including specialists) is conveyed, and the simplest contact channel will be suggested.

### Respondent selection and characteristics

The survey was designed to learn more about users of virtual clinical triage—their objectives, care intentions, needs and decisions taken, along with their demographic characteristics. Participating patient-users were anonymous/de-identified, and their final healthcare seeking action or behavior was not captured by this survey. Survey respondents represented a globally diverse population, as the triage engine is available in 20 languages. Although the application itself is available in 20 languages, the study included only respondents completing the interview in English. Only individuals at least 18 years old were eligible to use the triage engine and complete the survey, although parents/family members could triage on behalf of a minor child or elder.

### Data captured and analyses completed

The study was implemented, and the respondent data collected and analyzed through the Infermedica virtual triage engine, Symptomate, available at no cost to the general public. The analyses were completed by a multidisciplinary team of physicians, data scientists and business intelligence experts within Infermedica. All responses were totally anonymous with no identity captured, and all respondents explicitly consented to completion of the survey. Infermedica is fully compliant with GDPR. The survey captured respondent demographic data including age, sex and national location, plus self-reported clinical information including current symptoms, symptom severity and duration, disease risk factors and past medical history. Seven items comprised the survey, and the estimated completion time was 2–3 min. The survey data was analyzed by response item frequency with stratified analyses according to key demographic, care intent and user variables.

## Results

### Survey response volume and rate

There were 2,113 survey users of the triage engine during the defined study period of 8 weeks from February 15 to April 11, 2021, who opted in to complete the voluntary survey, with 93.9% of respondents using the triage engine on behalf of themselves rather than another individual. This is approximately 1% of all Symptomate virtual triage engine users during this period. No incentives were offered to respondents beyond the informational value delivered by the virtual triage engine itself.

### Virtual triage patient-user demographic characteristics

Seventy-eight percent of virtual triage users were female. With respect to age, 37% were 18–24 years old or younger, 28% were 25–44, 16% were 45–54, and 19% were 55 years or older. With respect to the nation where the survey was completed, 41.2% were from the U.S., 12.5% from the U.K., 9.1% from Canada, 5.6% from India, 3.8% from South Africa and the balance (27.2%) were from other nations.

### Patient motivation to utilize virtual triage

**Figure 1:**
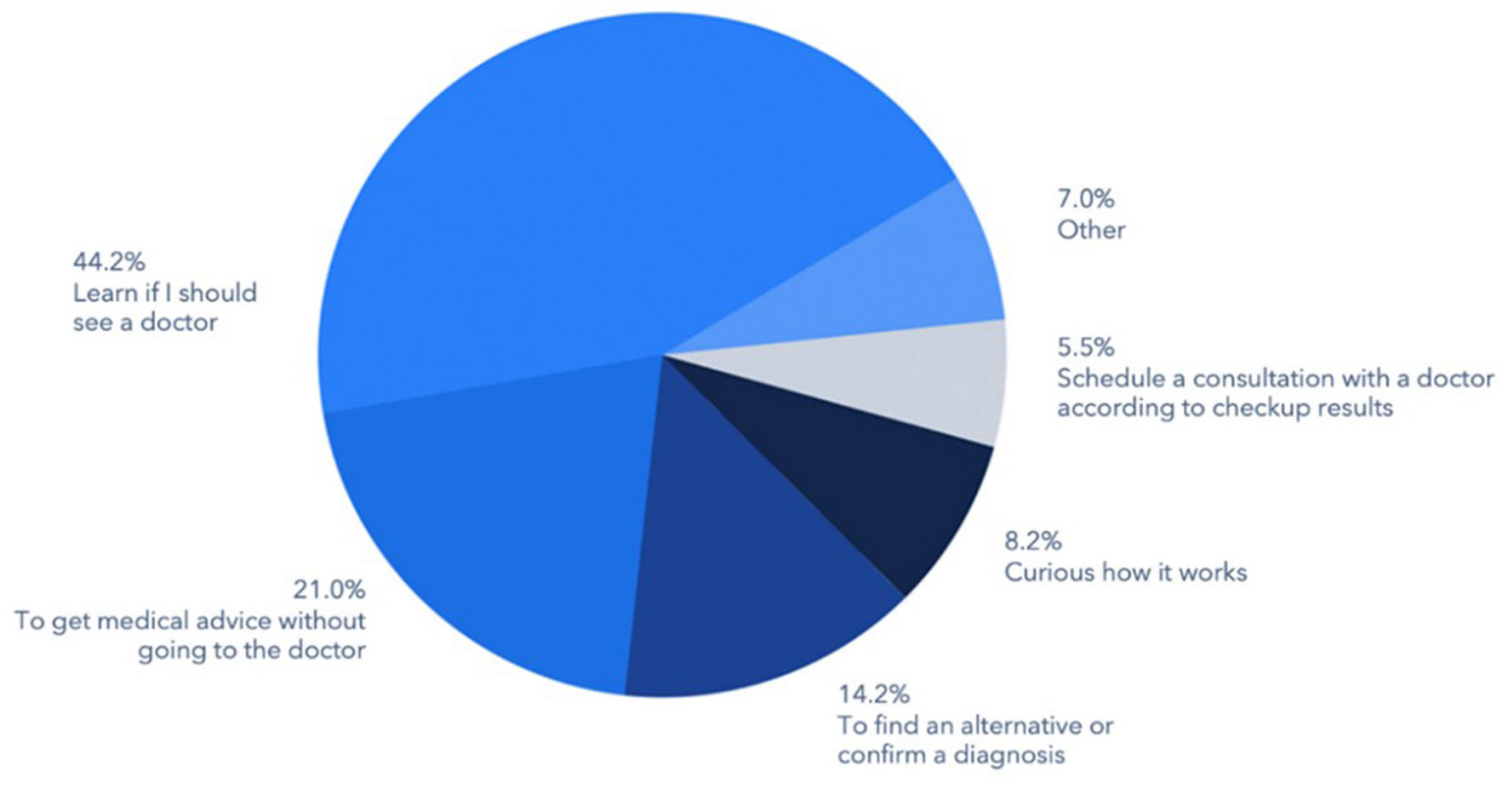
Motivation for utilizing a triage engine/symptom checker.

The most common motivations of consumer-patients for using virtual triage were to determine whether the individual needed to seek healthcare from a physician (44.2%), followed by a desire to secure medical advice without visiting a physician's office or other care delivery site (21.0%), or to confirm a diagnosis received from another source or to identify an alternative one (14.2%) (Figure 1). Another 8.2% cited curiosity as the reason for using virtual triage and 5.5% indicated that they were specifically seeking to schedule an appointment with a physician.

### Frequency of virtual triage utilization

**Figure 2:**
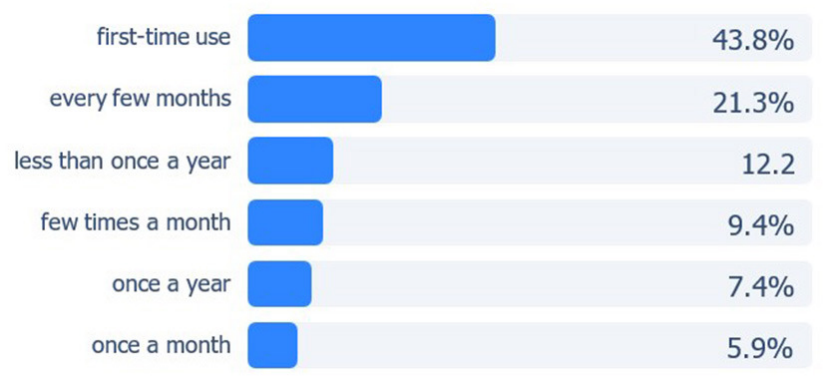
Frequency of virtual triage utilization.

Of 2,113 respondents, 43.8% were first time users of virtual triage. Over one-third (36.6%) utilized a triage engine at least once every few months or more often, 15.3% used one at least once per month or more frequently, 7.4% used virtual triage once per year and 12.2% less than once a year (Figure 2).

### Patient-user initial healthcare intent before engaging virtual triage

Pre-triage, 40.5% of patient-users did not know what level of healthcare they were planning to utilize. Another 33.9% stated they intended to seek a physician consultation, 23.7% intended to engage in self-care and 1.8% intended to seek care at an ED (Table 2).

**Table 2:** Pre-triage patient care seeking intent and disparity with triage output.

**Patient healthcare intent before virtual triage**	**Engage self-care**	**Seek physician consultation**	**Visit an ED**	**Did not know what level of care to engage**	**Triage care recommendation differed from patient pre-triage intent**	**Triage care recommendation same as patient pre-triage intent**
Respondents by pre-triage intent	23.7%	33.9%	1.8%	40.5%	—	—
Alignment of pre-triage intent with triage output	—	—	—	—	74.1% (27.9% will realign care intent to triage output)	25.9%

### Virtual triage engine determined distribution of healthcare recommendations

Virtual triage recommended 56.8% of patient-users consult a physician (33.1% within 24 hours and 23.7% when possible). Another 33.8% were advised to seek care at an ED (28.6% by self-transport and 5.2% by ambulance), and for 9.4% self-care was recommended.

### Disparity between patient pre-triage healthcare intent and triage engine output

Virtual triage provided information to support users in appropriately engaging self-care (managing symptoms at home typically), seeking a physician office or ambulatory consultation, or visiting an ED. In three-fourths of cases, virtual triage helped users decide what level of care to select and engage. Among 74.1% of respondents, the resulting triage recommendation for care was different than their healthcare intentions before virtual triage, including those that did not know or have an intent, and in 25.9% the patient's pre-triage intention matched the recommendation generated by virtual triage (Table 2).

### Impact of virtual triage on patient healthcare seeking intent

Virtual triage users were asked whether they intended to follow the guidance provided by the virtual triage engine, and 27.9% of users stated that they would follow triage recommendations if different than their initial care intent.

Of patients for whom virtual triage recommended engaging self-care, 69.5% of patients indicated that they would do so; 19.7% stated they would still seek a physician visit; 1.0% stated they would go to an ED; and 9.8% remained uncertain of the care they would pursue (Table 3).

**Table 3:** Impact of virtual triage on patient care seeking behavior.

		**Patient Care Intent After Virtual Triage**		
	Engage in Self-Care	Seek Physician Outpatient Consultation	Visit an Emergency Department	Uncertain of Care Path Before Triage
				40.5%
Virtual Triage Care Recommendation				Uncertain of Care Path After Triage
Engage Self-Care	69.5%	19.7%	1.0%	9.8%
Seek Physician Consultation	20.0%	66.6%	2.2%	11.2%
Visit an Emergency Department	24.2%	54.6%	12.0%	9.2%
Total				30.2%

Of patients for whom virtual triage recommended consulting a physician, 66.6% stated that they would do so while 20.0% stated they would engage only self-care, 2.2% intended to go to an ED while 11.2% remained uncertain of their care course (Table 3).

For patients for whom triage recommended visiting an emergency department, 12.2% indicated they would comply while 54.6% stated they would instead consult a physician, 24.2% would remain at home, and 9.2% remained unclear about their care intent (Table 3).

**Figure 3:**
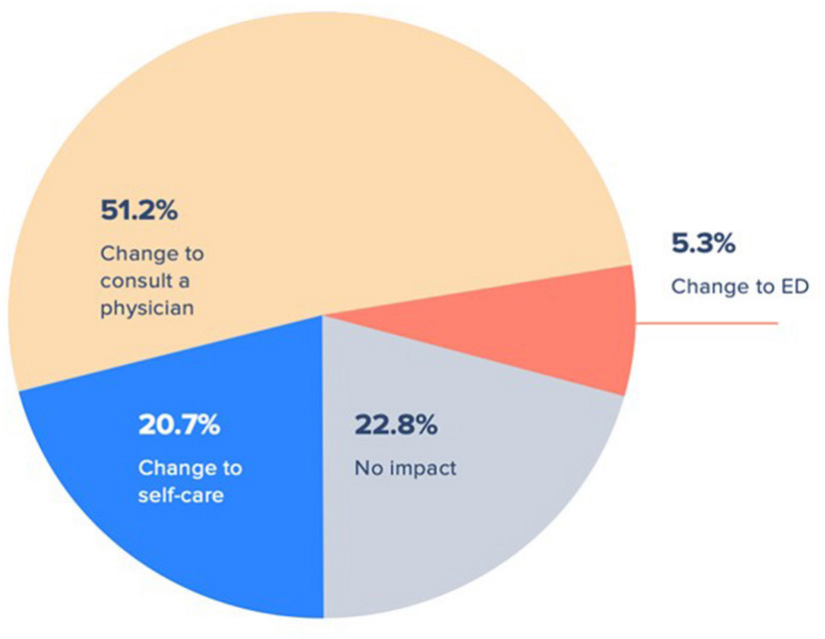
Changes in patient care seeking intent after completing virtual triage.

Across all care recommendations generated from virtual triage, individuals who remained uncertain of the care they would pursue after virtual triage were largely the same group of individuals who did not know what care they intended to pursue before virtual triage. The total size of this group decreased by 25.4% from 40.5% pre-triage to 30.2% post-triage. Over three-fourths of respondents declared that after using virtual triage they were more likely to change their mind regarding the acuity level of their care (Figure 3). Of these, 51.2% changed their intended healthcare seeking to consulting a physician, 5.3% changed to pursuing care by visiting an emergency department, and 20.7% altered their care plan to instead engage self-care.

### Referral to telemedicine and virtual healthcare

**Figure 4:**
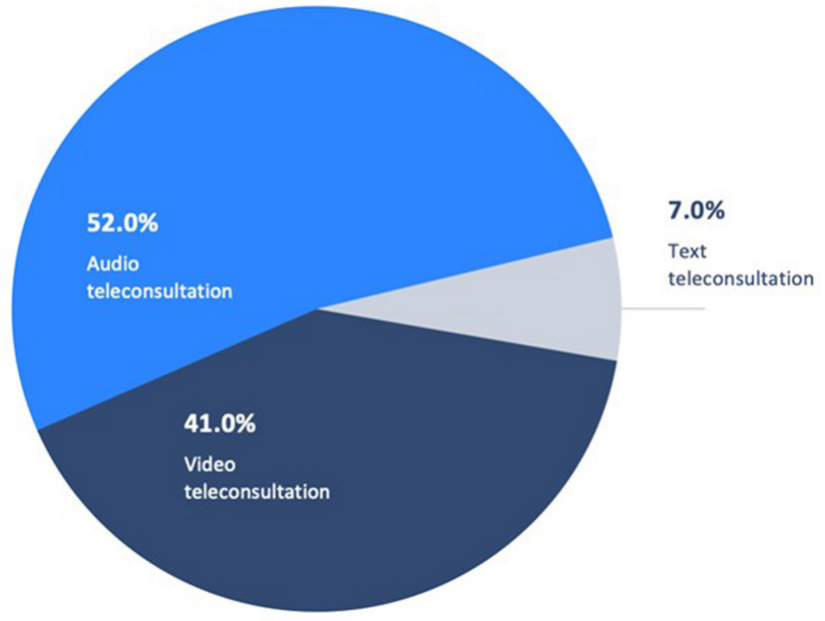
Teleconsultation vehicle recommended by virtual triage.

Following use of virtual triage, a greater number of respondents initially seeking an in-person physician consultation were recommended to instead use a remote/virtual means of engagement or communication channel. Prior to use of virtual triage, 16% of patients intended to use a telemedical/virtual consultation, and following triage 28% of patients did, an increase of 42.9%. Video or audio teleconsultation was recommended for a large majority of these individuals based on triage clinical assessment (Figure 4).

### Patient experience and satisfaction with virtual triage

Patient-user experience and satisfaction with virtual triage was high, with 53.3% stating that they were highly likely to use the application again and 27.4% that they were likely to, or a total of 80.1% of patients indicating substantial overall satisfaction with the experience and value delivered. Only 5.5% of patient-users stated that they were unlikely or very unlikely to use virtual triage again. The fact that 43.8% of respondents were first time as opposed to returning users suggests that the value conveyed by virtual triage is discrete and does not require repeated use to deliver value to patients during a particular illness episode. It also suggests that virtual triage may be a useful method to engage more patients digitally. Of those who did not know what care level to seek prior to digital triage, 30.3% indicated that they found the guidance they needed through virtual triage.

### Streamlining and accelerating appropriate medical care specialty referral

**Figure 5:**
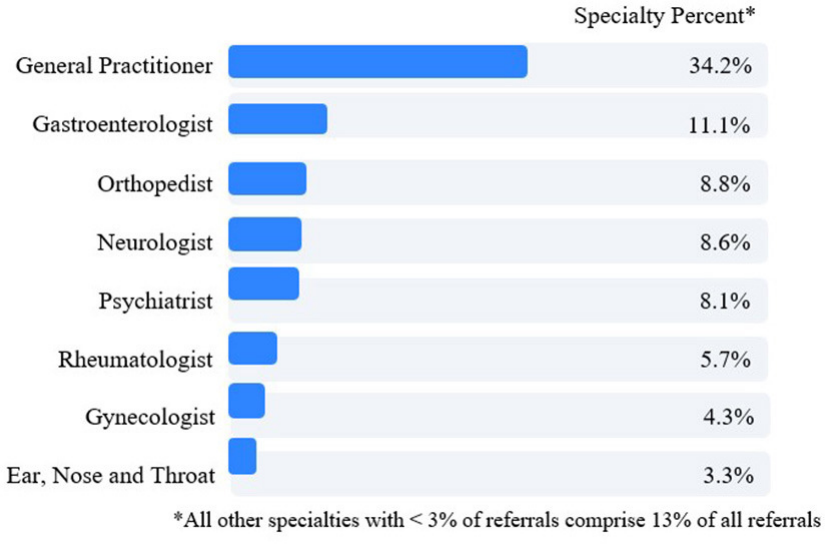
Virtual triage care referral recommendation by physician specialty.

With respect to referral for physician consultation and care, virtual triage referred 56.8% of patient users to a physician care level. Of patients referred to physician consultation, 34.2% were directed to a general/family medicine practitioner (Figure 5). The next four most frequent specialists to whom patients were referred were gastroenterologists (11.1%), orthopedists (8.8%), neurologists (8.6%) and psychiatrists (8.1%).

### Cross-tabulation of patient-user demographics and virtual triage experience and use patterns

Correlations between patient-user demographics and key metrics and patterns of virtual triage use were examined, with the following chi^2^ results, all significant at the *p* < 0.001 level.

### Age and frequency of triage use, pre-triage healthcare intent, intent to follow triage care recommendation and triage experience satisfaction

Regarding age and frequency of virtual triage use, respondents aged 75 or older were more often first time users. Those aged 45–59 used virtual triage once a year or less frequently. Respondents ages 30–44 and 12–17 more often used virtual triage once every few months, and those ages 18–29 use virtual triage monthly. With respect to the relationship between age and pre-triage healthcare intent, patient-users age 75 years and older more often intended to go to an emergency department or engage self-care; to individuals aged 45–59 years more often did not know or were uncertain about what care plan to pursue; and those 12–17 more often indicated they plan to consult a physician. With respect to age and intent to follow the post-triage care recommendation, individuals 75 and older more often stated they will go to the emergency department when recommended by virtual triage; those aged 45–59 more often indicated they would see a physician on an outpatient basis if recommended by triage; individuals aged 18–29 stated they would engage self-care more often if triage recommended; and those 12–17 more often indicated that the triage recommendation had no impact on them. With regard to patient-user satisfaction with virtual triage and age, respondents aged 75 and older stated they were very unlikely and those 12–17 were unlikely to use triage again, respectively; those 60–74 more often indicated that they would likely use virtual triage again; those 40–59 that they were very likely to use virtual triage again.

#### Gender and frequency of triage use, pre-triage healthcare intent, intent vs. triage care recommendation, and triage care recommendation

Men were more often first time users of virtual triage than women, and women more often indicated that they use virtual triage one a year, once a month or once every few months than men. With respect to pre-triage healthcare intent, men more frequently planned for self-care, to consult a physician on an outpatient basis or to visit an emergency department, while women more frequently did not know what care to pursue. Regarding pre-triage intent vs. care acuity level recommended by virtual triage, men more frequently matched the triage recommended level of care acuity, and also, to over-triage to a possibly higher level of care acuity than needed, while women tended to under-triage and to plan care engagement at a lower level of acuity than recommended by virtual triage. In terms of the care acuity recommendation conveyed by virtual triage, men were more frequently recommended to engage self-care, consult a physician within 24 hours or to call an ambulance for transport to an emergency department; while women were more frequently advised to seek outpatient physician consultation or to visit an emergency department.

## Discussion

This study demonstrates that virtual triage is useful to individuals who do not know if their symptoms warrant medical attention or self-care, what next steps should be pursued, and the care level acuity needed. A substantial percentage of patients struggle to navigate the complexity of healthcare services. Virtual triage engaged at the beginning of an illness episode can assist patient-users by conveying guidance in the selection, access and use of different levels/sites of healthcare service acuity. We found 40.5% of triaged patients seeking medical information online did not know what kind of healthcare they needed—self-care, physician consultation or ED visit. This uncertainty produces two challenges—patients in need of services hesitating to consult with a healthcare professional, and/or visiting an inappropriate type or acuity level of care facility.

Virtual triage assesses and analyzes patients' health status and clinical symptomatology before recommending a specific kind of healthcare, and thus delivers care value in conveying appropriate preliminary medical information and guidance. Over two-thirds of survey respondents stated their motivation to use virtual triage was either to determine if a physician consultation was needed or to secure medical guidance without consultation. Not surprisingly, virtual triage users tend to be younger (63% below age 45), with women apparently more amenable to virtual triage than men. Given the novel nature of virtual triage, it is also expected that almost half were first time users, although over one-third use virtual triage at least once every few months or more frequently. That three in four triage users had a pre-triage care intent that differed from what virtual triage recommended based on clinical presentation is a striking finding requiring further evaluation, and the recommended acuity of care and specialty referral require validation.

In particular, the large post-triage referral to ED care warrants clinical validation that this care acuity was in fact clinically imperative, or if in the pursuit of high triage sensitivity and safety to avoid not detecting acute care needs virtual triage is reducing specificity by over-referring to the ED. Emergency ambulance was recommended in 5.3% of the cases, of which 23% had chest pain as one of the initial symptoms and 20% had dyspnea, suggesting that a large proportion of these referrals were appropriate. Emergency care was recommended by virtual triage in 29.4% of the cases, of which 15% reported chest pain as one of the initial symptoms and 16% reported dyspnea. Among patient-users recommended emergency care, leading diagnostic possibilities identified were asthma exacerbation (4%), COVID-19 (2.9%), and pneumonia (2.3%). Among those recommended emergency ambulance, the leading diagnostic possibilities identified were myocardial infarction (19.6%), pulmonary embolism (9.8%), and subarachnoid hemorrhage (7.1%). These figures support the appropriateness of referral to an ED.

The data captured and presented in this report is limited by the demographics of the virtual triage user population and thus generalizability. The respondent sample was predominantly female, skewed to younger age strata, and almost two-thirds completed triage from the US, UK or Canada. This population may behave differently in healthcare seeking and use of virtual health than other population segments. The data is also limited by the fact that the virtual triage engine can identify the nation from which triage was completed and language used, but there is no validation that an English speaker in Canada, for example, is not an American living in or visiting the US. Another current limitation of the triage engine and the interpretation of the data presented is that validation of what patient-users expressed as their healthcare use intent was not confirmed with information on what the patient actually did following their use of virtual triage. Stated intention may be subject to various biases, including social desirability, and thus may not be uniformly reliable.

The percentage of patients using the triage application who were recommended telemedicine/virtual engagement of care instead of an in-person visit almost doubled, and this has significant implications and value for facilitating patient engagement of these rapidly evolving and expanding care delivery vehicles. Educating patients and providing options for audio, video, or text teleconsultation broadens patients' perspectives and opens the door for digital healthcare adoption and associated reductions in avoidable, unnecessary acute in-person care where clinically appropriate, effective and safe.

Failure to early detect and treat illness can lead to avoidable chronic health problems, existing disease exacerbation, and avoidable healthcare utilization and costs. Virtual triage successfully redirected 27.9% of users who initially planned to seek an inappropriate level of care acuity and doubled the percentage of patients amenable to telemedicine and virtual health engagement. Virtual triage can analyze patients' clinical presentation and status before engaging in healthcare seeking and in-person consultations and are a tool for helping patients pursue the appropriate level of healthcare acuity based on their symptom presentation and medical history (12). Moreover, the evidence provided in this survey of virtual triage patient-users indicates that virtual triage is an engaging, effective and powerful vehicle for health systems to deliver value to patients beyond the four walls of the hospital, and thus to align patients and their families more systematically across the full lifecycle of their healthcare needs and as patients actually experience them. Patient-users of virtual triage were highly satisfied with the virtual triage patient experience and triage output, with 80% indicating that they will use triage recurrently in the future, a result similar to the findings of a systematic review of 27 prior reports on symptom checkers (13).

## Data availability statement

The original contributions presented in the study are included in the article/supplementary material, further inquiries can be directed to the corresponding author/s.

## Ethics statement

Ethical review and approval was not required for the study of human participants in accordance with local legislation and institutional requirements. Written informed consent from the patients/participants was not required to participate in this study in accordance with national legislation and institutional requirements. Patient-users opted in voluntarily in order to participate in the survey.

## Author contributions

GG led the team effort to complete a manuscript, completed part of the data analyses, integrated all source content, and wrote the manuscript. PO led the multidisciplinary, cross-department teams that designed and implemented the virtual engine, and the survey. TP contributed to the analyses and writing of the manuscript, and helped implement and refine the triage technology. AK-K completed biostatistical analyses. JJ and NM contributed to the analyses, interpretation of the data, and the writing of the manuscript. AM, AK, and PK contributed to the analyses and interpretation of the data. All authors contributed to the article and approved the submitted version.
